# Development and psychometric validation of a knowledge–attitude–practice questionnaire on geriatric foot care among clinical nurses

**DOI:** 10.1371/journal.pone.0356470

**Published:** 2026-08-17

**Authors:** Jing Yu, YouFen Huang, Ying Zeng, Ling Zeng, Ling Li

**Affiliations:** 1 Department of Geriatrics, Chongqing Academy of Medical Sciences, Chongqing General Hospital, Chongqing University, Chongqing, China; 2 Department of Nursing, Guizhou Provincial People’s Hospital, Guiyang, Guizhou, China; Shandong University, CHINA

## Abstract

**Objective:**

To develop a Knowledge–Attitude–Practice (KAP) questionnaire on geriatric foot care for clinical nurses in China and to evaluate its reliability and validity.

**Methods:**

The questionnaire was developed based on the Knowledge–Attitude–Practice (KAP) framework through a literature review, semi-structured interviews, and two rounds of Delphi expert consultation. After pilot testing, a cross-sectional survey using convenience sampling was conducted among 324 clinical nurses from two tertiary general hospitals in Guizhou Province and Chongqing Municipality, China. Item analysis, exploratory factor analysis (EFA), confirmatory factor analysis (CFA), and assessments of reliability and validity were performed to examine the psychometric properties of the questionnaire.

**Results:**

The final questionnaire comprised 48 items across three dimensions: knowledge, attitude, and practice. Exploratory factor analysis yielded a cumulative variance contribution of 58.314%. Confirmatory factor analysis demonstrated satisfactory model fit (χ²/df = 1.489, GFI = 0.912, AGFI = 0.932, CFI = 0.948, and RMSEA = 0.055). The scale-level content validity index (S-CVI) was 0.93. The overall Cronbach’s alpha coefficient was 0.933, and both split-half reliability and test–retest reliability coefficients exceeded 0.80.

**Conclusion:**

The questionnaire demonstrated satisfactory psychometric properties, with acceptable reliability and validity. It may be useful for assessing clinical nurses’ knowledge, attitudes, and practices regarding geriatric foot care and for identifying educational needs in this area.

## Introduction

Older adults commonly experience foot problems arising from age-related changes and comorbid conditions that affect foot biomechanics, physiology, and morphology [1]. As the population in China continues to age, foot disorders have become increasingly common among older adults, with reported prevalence rates ranging from 71% to 87% [2]. Declines in flexibility, vision, and fine motor function associated with aging may compromise the ability of older adults to perform adequate foot self-care [3,4]. In addition to affecting daily functioning, foot problems have been linked to frailty, reduced mobility, falls, disability, and mortality, ultimately impairing quality of life and well-being [5].

Despite their frequency and potential consequences, foot problems in older adults remain insufficiently addressed in routine nursing care [6]. Clinical nurses may have limited opportunities for education and training in foot care, which could affect their ability to provide assessment, prevention, and patient education. Several instruments related to foot care have been developed and validated; however, most were designed for diabetic populations and focus primarily on patient self-management behaviors or disease-specific outcomes. Such instruments may not adequately address the competencies required for geriatric foot care nursing. Given the multifactorial nature of foot problems in older adults, existing tools may not adequately capture nurses’ knowledge, attitudes, and practices related to geriatric foot care.

Although nurses play a key role in the assessment, prevention, and management of foot problems among older adults, research examining nurses’ knowledge, attitudes, and practices in this area remains scarce. To our knowledge, no validated instrument specifically developed to assess Chinese clinical nurses’ knowledge, attitudes, and practices regarding geriatric foot care has been reported. The lack of such a measure limits the ability to identify educational needs and to evaluate competencies related to geriatric foot care.

Providing foot care to older adults requires nurses to recognize common age-related foot problems, appreciate the importance of preventive care, and incorporate appropriate assessment and educational practices into routine care. These competencies correspond closely to the domains of knowledge, attitude, and practice. The KAP framework was therefore considered an appropriate conceptual basis for the development of the present questionnaire. Accordingly, this study aimed to develop and validate a KAP questionnaire to assess clinical nurses’ knowledge, attitudes, and practices regarding geriatric foot care.

## 1. Methods

### 1.1 Questionnaire development

#### 1.1.1 Item generation.

The initial item pool was developed based on the KAP theoretical framework through a combination of literature review and semi-structured interviews. A systematic literature search was conducted in PubMed, Embase, Web of Science, the Cochrane Library, China National Knowledge Infrastructure (CNKI), Wanfang Data, VIP Database, and the Chinese Biomedical Literature Database. Search terms included concepts related to older adults, foot health, and nursing care, such as elderly, aged, foot, foot care, foot disorders, nursing, and self-care. Existing questionnaires [7,8], clinical guidelines [9], and expert consensus documents [10] were reviewed to inform item development.

In parallel, semi-structured interviews were conducted with 13 clinical nursing experts specializing in geriatric care at a tertiary hospital in Guizhou Province. Interview data were analyzed using Colaizzi’s seven-step phenomenological method to identify key themes relevant to geriatric foot care. Based on the findings from the literature review and qualitative analysis, the research team iteratively discussed, refined, and revised the items. This process resulted in a preliminary questionnaire comprising 62 items across three dimensions—knowledge, attitude, and practice—designed to assess clinical nurses’ competence in geriatric foot care.

#### 1.1.2 Delphi expert consultation.

A two-round Delphi expert consultation was conducted to screen and refine the preliminary questionnaire. Experts were recruited from the fields of geriatric nursing, foot and ankle care, wound and ostomy care, nursing education, and clinical practice. Eligibility criteria included at least 10 years of professional experience, a bachelor’s degree or higher, an intermediate or senior professional title, and voluntary participation. Experts were asked to rate the importance of each item using a 5-point Likert scale (1 = very unimportant to 5 = very important) and to provide suggestions for item modification, deletion, or addition. Items were retained based on the following criteria: mean importance score ≥ 4.0, coefficient of variation ≤ 0.25, and expert agreement rate ≥ 75%. After two rounds of consultation, expert opinions reached consensus, and a pretest version of the questionnaire comprising 55 items across three dimensions (knowledge, attitude, and practice) was finalized.

#### 1.1.3 Pilot testing.

A pilot test was conducted in April 2022 among 20 clinical nurses working in geriatric care at a tertiary general hospital in Guizhou Province. Participants completed the questionnaire in a face-to-face setting and were asked to provide feedback on item clarity, readability, and feasibility. The average completion time was recorded. Based on participants’ feedback, minor wording revisions were made to improve clarity, while no items were added or removed. The results indicated that the questionnaire was easy to understand and feasible for formal administration.

### 1.2 Item analysis and psychometric evaluation

#### 1.2.1 Participants and data collection.

From 01 April to 31 May 2022, clinical nurses working in geriatric care units at two tertiary general hospitals in Guizhou Province and Chongqing, China, were recruited through convenience sampling. Inclusion criteria were registered nurse licensure, at least two years of clinical experience, and voluntary participation with written informed consent. Nurses who were not registered at the study hospitals, were trainees, or were on long-term sick or maternity leave (≥3 months) were excluded. This study was approved by the Ethics Committee of Guizhou Provincial People’s Hospital (Approval No. 2021−88). Written informed consent was obtained from all participants prior to data collection. Participation was voluntary, and participants could withdraw from the study at any time without penalty. All data were anonymized before analysis to ensure confidentiality. The study was conducted in accordance with the principles of the Declaration of Helsinki. Sample size was determined based on the number of questionnaire items and requirements for factor analysis. Following Kendall’s recommendation, a sample size of 5–10 participants per item was considered appropriate [11], with a minimum of 100 participants required for exploratory factor analysis [12]. Allowing for a 10% attrition rate, a minimum sample size of 305 was calculated.

The questionnaire consisted of three dimensions—knowledge (26 items), attitude (9 items), and practice (20 items). Knowledge items were scored dichotomously (“yes,” “no,” or “unknown”), with correct responses scored as 5 points and incorrect or unknown responses scored as 0 (range: 0–130). Attitude and practice items were rated on 5-point Likert scales, with higher scores indicating more positive attitudes and better practices. The total score ranged from 29 to 275, with higher scores reflecting higher overall levels of knowledge, attitudes, and practices related to geriatric foot care. Data were collected using an online survey platform, and statistical analyses were performed using SPSS version 26.0.

#### 1.2.2 Item analysis.

Item analysis was conducted using the critical ratio method and item–total correlation analysis. Total scores were ranked, and the upper and lower 27% of respondents were compared using independent-samples t tests. Items with t values greater than 3.0 and P < 0.05 were retained. Pearson correlation coefficients between individual items and the total questionnaire score were calculated, with coefficients ≥ 0.30 considered acceptable for item retention.

#### 1.2.3 Validity assessment.

Content validity was evaluated using the item-level content validity index (I-CVI) and scale-level content validity index (S-CVI). Based on the second round of expert consultation, the I-CVI was calculated as the proportion of experts rating an item as 4 or 5, with values ≥ 0.75 considered acceptable. The S-CVI was calculated as the average of all I-CVI values, with values > 0.90 indicating good content validity [13].

Construct validity was examined using exploratory factor analysis (EFA) and confirmatory factor analysis (CFA). Exploratory factor analysis was conducted using principal component analysis (PCA) with varimax rotation. PCA was selected as an exploratory approach to examine the underlying structure of the newly developed questionnaire and to facilitate item reduction during the initial stages of scale development. Sampling adequacy was assessed using the Kaiser–Meyer–Olkin (KMO) measure and Bartlett’s test of sphericity, with KMO values > 0.60 considered acceptable [14]. Items with factor loadings < 0.40, cross-loadings (difference < 0.15), or fewer than three items per factor were removed [15]. A cumulative variance contribution of at least 40% and factor loadings > 0.40 were considered acceptable [16]. CFA was conducted using maximum likelihood estimation. Model fit was evaluated using χ²/df, the goodness-of-fit index (GFI), adjusted goodness-of-fit index (AGFI), comparative fit index (CFI), and root mean square error of approximation (RMSEA). Values of χ²/df < 2.0, GFI > 0.85, AGFI > 0.90, CFI > 0.90, and RMSEA < 0.08 indicated acceptable model fit [17].

#### 1.2.4 Reliability assessment.

Reliability was assessed using internal consistency, split-half reliability, and test–retest reliability. Internal consistency was evaluated using Cronbach’s alpha, with values ≥ 0.70 considered acceptable [18]. Split-half reliability was assessed by dividing the questionnaire into odd- and even-numbered items and calculating the correlation between the two halves. Test–retest reliability was evaluated in a subsample of 20 nurses who completed the questionnaire again after a two-week interval, using Spearman correlation coefficients, with values > 0.70 indicating satisfactory stability [13].

## 2. Results

### 2.1 Results of the Delphi expert consultation

A total of 22 experts from hospitals and academic institutions in Chongqing, Guizhou, Shanghai, Guangzhou, Tianjin, Jiangsu, and Hunan participated in the Delphi consultation. The experts represented multiple disciplines, including geriatric medicine and nursing, foot and ankle care, wound and ostomy care, and nail disorders. Among the experts, 19 were female and 3 were male, with a mean age of 40.77 ± 5.76 years. Seven experts held a master’s degree and three held a doctoral degree. Ten experts had senior associate professional titles and two had senior professional titles. The mean length of professional experience was 17.45 ± 8.17 years.

Two rounds of expert consultation were conducted, with effective response rates of 91.7% and 100%, respectively. The authority coefficients for the two rounds were 0.871 and 0.875, with judgment basis coefficients of 0.932 and 0.941 and familiarity coefficients of 0.809 in both rounds, indicating high expert authority and engagement. Kendall’s coefficients of concordance were 0.209 and 0.227 for the two rounds, respectively (both P < 0.05), demonstrating acceptable agreement among experts. In the first round, mean item importance scores ranged from 3.41 to 4.91, with coefficients of variation between 0.06 and 0.39 and expert agreement rates ranging from 45.4% to 100%. Based on predefined criteria and expert feedback, seven items were removed and eleven items were revised. In the second round, mean importance scores ranged from 4.00 to 4.95, coefficients of variation ranged from 0.04 to 0.25, and expert agreement rates ranged from 77.3% to 100%. No further modifications were suggested, indicating consensus had been achieved. The resulting preliminary questionnaire comprised 55 items across three dimensions: knowledge, attitude, and practice.

### 2.2 Pilot testing results

A total of 20 clinical nurses participated in the pilot test, and all questionnaires were completed and returned, yielding a response rate of 100%. The completion time ranged from 5 to 15 minutes. Overall, participants reported that the questionnaire items were clear, understandable, and easy to complete. Based on participant feedback, minor wording refinement was made to one item in the practice dimension to improve clarity, while no items were added or deleted. The pilot test results indicated that the questionnaire demonstrated good feasibility for formal administration.

### 2.3 Item analysis results

A total of 336 questionnaires were distributed, and 324 valid questionnaires were returned, yielding an effective response rate of 96.4%. Item analysis was performed using the critical ratio (CR) method, item–total correlation analysis, internal consistency testing, and exploratory factor analysis. Using the critical ratio method, items with CR values < 3.0 and P > 0.05 were identified and considered for removal. Item–total correlation analysis showed that several items had correlation coefficients < 0.30. Internal consistency analysis indicated that removal of these items resulted in an increase in the overall Cronbach’s alpha coefficient. Exploratory factor analysis further identified items with factor loadings < 0.40. Based on the predefined criteria, items that failed to meet at least two of the above criteria were removed. After item reduction, four items were deleted from the knowledge dimension, and the remaining items were retained for subsequent analyses.

### 2.4 Validity results

#### 2.4.1 Construct validity.

A second exploratory factor analysis (EFA) was conducted on the remaining 51 items after item analysis. The Kaiser–Meyer–Olkin (KMO) measure and Bartlett’s test of sphericity indicated that the data were suitable for factor analysis. Principal component analysis with varimax rotation was performed, and three factors were extracted in accordance with the KAP theoretical framework. During this process, three knowledge items with factor loadings < 0.40 were identified and removed. A third EFA was subsequently performed on the remaining 48 items. The KMO value was 0.940, and Bartlett’s test of sphericity was significant (χ² = 14,759.458, P < 0.001), confirming sampling adequacy. Three factors were extracted, accounting for a cumulative variance contribution of 58.314%, which exceeded the recommended threshold of 40%. All retained items demonstrated factor loadings greater than 0.40. Detailed factor loadings are presented in Table 1. The final questionnaire consisted of 48 items across three dimensions. Factor 1 comprised 19 items representing knowledge of geriatric foot care, including foot skin care, nail care, structural deformities, and footwear selection. Factor 2 included 9 items reflecting attitudes toward geriatric foot care. Factor 3 comprised 20 items representing geriatric foot care practices across similar domains. Confirmatory factor analysis indicated good model fit, with χ²/df = 1.489, goodness-of-fit index = 0.912, adjusted goodness-of-fit index = 0.932, comparative fit index = 0.948, and root mean square error of approximation = 0.055.

**Table 1:** Factor loadings of items after rotation in the third exploratory factor analysis.

Item	Factor		
	3	2	1
P11 I guide older adults to perform foot self-examinations	0.900		
P10 I instruct older adults with conditions such as diabetes to wash their feet properly and follow precautions	0.892		
P15 I guide and assist older adults in using foot orthoses or supportive footwear when necessary	0.872		
P13 I recommend that older adults with diabetes or osteoarthritis attend podiatry clinics for foot health assessment	0.869		
P12 When serious foot problems are identified, I seek multidisciplinary collaboration	0.855		
P5 I assess the toenail condition of older adults	0.853		
P16 I advise older adults not to wear socks with excessive seams or holes	0.842		
P14 I guide older adults in selecting appropriate footwear and socks, including material, width, depth, and heel height	0.832		
P6 I guide or assist older adults in trimming toenails correctly (straight cut, level with the toe, smoothing edges with a file)	0.827		
P2 I guide or assist older adults in maintaining foot hygiene, including drying between toes after washing	0.823		
P17 I encourage older adults to perform foot function exercises	0.820		
P20 I regularly provide foot care health education to older adults or their caregivers	0.818		
P4 I guide or assist older adults in managing corns or calluses appropriately	0.813		
P8 When older adults have fungal nail infections, I instruct them to use antifungal medication as prescribed	0.808		
P3 I guide or assist older adults in applying moisturizer after foot washing to prevent dryness	0.803		
P7 When mild ingrown toenails occur, I know how to place cotton under the nail edge to relieve discomfort	0.784		
P1 I assess the foot skin of older adults, including between toes and heels	0.767		
P18 I encourage older adults to stand after prolonged sitting (≥1 hour)	0.751		
P9 When examining feet, I assess pedal pulses, skin temperature, and sensation	0.745		
P19 I encourage older adults, especially those with diabetes, to stop smoking	0.742		
A7 I believe that providing foot care health education to older adults is necessary		0.948	
A5 I believe that shoe size assessment and footwear selection are essential for geriatric foot care		0.939	
A8 I believe that foot care can promote foot health in older adults		0.931	
A9 I believe that disease characteristics and clinical symptoms must be considered when providing foot care		0.931	
A4 I believe that older adults with chronic diseases (e.g., diabetes) are priority populations for foot care		0.909	
A3 I believe that foot pain and infections have the greatest impact on older adults		0.900	
A6 I believe that geriatric foot health management requires multidisciplinary collaboration		0.891	
A1 I believe that nurses play an important role in geriatric foot health management		0.888	
A2 I believe that the foot care needs of older adults are currently unmet		0.875	
K18 For patients with rheumatoid arthritis, footwear should provide adequate toe box height and width			0.671
K15 Collapse of any of the three foot arches may result in foot problems			0.653
K7 The correct method of toenail trimming is to cut off the corners of the nail			0.647
K12 Fungal infections of the nail or skin should be treated promptly to prevent ulcers and secondary infections			0.631
K25 Five-toe socks can reduce interdigital maceration and prevent tinea pedis			0.618
K23 Adequate toe space requires the shoe interior to be 1–1.5 cm longer than the longest toe			0.596
K13 Flatfoot should be managed with appropriately sized shoes that provide arch support and heel stability			0.584
K22 Feet tend to become wider and longer with age, making regular shoe size assessment important			0.573
K24 Shoes with shallow toe boxes can prevent toe deformities			0.560
K17 Routine foot soaking is an appropriate skin care practice for patients with diabetes			0.538
K16 The feet of patients with diabetes should be assessed daily			0.535
K19 Reduced skin temperature in one foot may indicate vascular obstruction			0.531
K26 Corns and calluses are not influenced by the type of socks worn			0.493
K1 Foot health assessment is required for older adults during hospitalization			0.490
K6 Toenails should preferably be trimmed before washing or when the feet are dry			0.484
K9 Cutting toenails too short may lead to ingrown toenails			0.476
K21 Older adults are advised to wear soft-soled shoes to prevent falls			0.451
K2 Corns and calluses are caused by prolonged abnormal pressure or friction			0.428
K4 Dry foot skin requires daily use of moisturizers, including between the toes			0.424

#### 2.4.2 Content validity.

Content validity was evaluated based on ratings from 22 experts in the second round of the Delphi consultation. The item-level content validity index (I-CVI) values ranged from 0.78 to 1.00. The scale-level content validity index (S-CVI/Ave) was 0.93, indicating good content validity of the questionnaire.

### 2.5 Reliability results

The overall internal consistency of the questionnaire was high, with a Cronbach’s alpha coefficient of 0.933. The Cronbach’s alpha coefficients for the three dimensions ranged from 0.877 to 0.978. Split-half reliability analysis showed an overall coefficient of 0.944, with coefficients of 0.874 and 0.870 for the two halves. Test–retest reliability was assessed in a subsample of 20 nurses over a two-week interval, yielding an overall correlation coefficient of 0.814. The test–retest reliability coefficients for the three dimensions ranged from 0.717 to 0.731.

## 3. Discussion

### 3.1 Significance of developing a KAP questionnaire on geriatric foot care for clinical nurses

Population aging has emerged as a major global public health challenge, and promoting health among older adults is central to achieving the goal of healthy aging. Among age-related health concerns, foot health plays a critical role in maintaining functional independence and quality of life. However, previous studies have shown that many older adults lack adequate knowledge, skills, and capacity for foot self-care [19], and foot health issues remain insufficiently emphasized in nursing practice [6]. Although routine nursing care standards incorporate foot hygiene and nail care as essential components of basic care, systematic assessment of clinical nurses’ knowledge, attitudes, and practices related to geriatric foot care has been limited. In addition, validated instruments designed to quantify these dimensions within nursing practice are lacking, particularly in the Chinese context.

Guided by the KAP framework, this study developed a questionnaire comprising 48 items across three dimensions—knowledge, attitude, and practice—to assess clinical nurses’ knowledge, attitudes, and practices regarding geriatric foot care. The questionnaire demonstrated satisfactory psychometric properties, with acceptable reliability and validity. It may be useful for identifying educational needs and informing future research on geriatric foot care competencies among clinical nurses.

### 3.2 Scientific rigor of questionnaire development

The geriatric foot care KAP questionnaire for clinical nurses was developed within a well-established behavior change framework and in strict accordance with standard procedures for scale development, ensuring scientific rigor, validity, and clarity. Initial item generation was informed by a comprehensive review of domestic and international literature, synthesizing evidence relevant to geriatric foot care into a preliminary item pool. Semi-structured interviews were then conducted to refine themes and enhance contextual relevance, resulting in an initial pool of 62 items. Item refinement proceeded through two rounds of Delphi expert consultation involving 22 experts, during which items were systematically evaluated and revised to produce a pretest version of the questionnaire. A pilot study was subsequently conducted to assess item clarity, comprehensibility, and completion time, leading to minor wording adjustments and confirmation of feasibility for clinical use. Following formal data collection, item analysis and psychometric testing were applied to further refine the scale based on predefined statistical criteria, yielding a final questionnaire with robust measurement properties.

Overall, the stepwise and iterative development process—integrating theoretical guidance, qualitative input, expert consensus, pilot testing, and quantitative validation—ensured comprehensive content coverage, clear item expression, a stable theoretical structure, and reliable measurement performance. These methodological features support the scientific soundness and reliability of the questionnaire for use in both research and clinical practice.

### 3.3 Psychometric properties and comparison with existing instruments

The psychometric evaluation of the geriatric foot care KAP questionnaire demonstrated satisfactory measurement performance across multiple indices. The final three-factor structure was supported by both exploratory and confirmatory factor analyses, with acceptable factor loadings and a cumulative variance contribution exceeding commonly recommended thresholds. Model fit indices from confirmatory factor analysis further indicated that the hypothesized structure was empirically supported, suggesting adequate construct validity and theoretical coherence. Reliability testing showed high internal consistency at both the overall scale and subscale levels, as well as acceptable split-half and test–retest reliability, indicating stable measurement performance over time. These findings are consistent with established psychometric standards for KAP-based instruments and support the questionnaire’s suitability for assessing clinical nurses’ competence in geriatric foot care.

Compared with existing instruments that primarily focus on foot health outcomes or patient self-care behaviors, the present questionnaire uniquely targets clinical nurses and integrates knowledge, attitudes, and practices within a single framework. While several international tools [20,21] assess foot care knowledge or behaviors in specific populations—such as patients with diabetes—few instruments comprehensively evaluate nursing-related foot care competence, particularly in the context of geriatric care. Moreover, some existing scales emphasize single dimensions or lack rigorous validation procedures, limiting their applicability in clinical education and quality improvement initiatives. By contrast, the current questionnaire was developed through a theory-driven, multi-method process and underwent systematic psychometric validation in a clinical nursing population. Its multidimensional structure allows for differentiated assessment of cognitive, attitudinal, and behavioral components, providing a more nuanced understanding of nursing competence in geriatric foot care. This feature enhances its potential utility for needs assessment, intervention evaluation, and research on nursing education and practice improvement.

### 3.4 Applicability and scope of use

The geriatric foot care KAP questionnaire demonstrated good acceptability in terms of item number, wording clarity, and response burden. In formal administration, nurses completed the questionnaire within 5–15 minutes, with no substantial comprehension difficulties reported, indicating good feasibility for routine use. By integrating knowledge, attitude, and practice dimensions, the instrument provides a multidimensional assessment of nurses’ competence in geriatric foot care. The questionnaire can be used to assess training needs, evaluate educational outcomes, and support research in clinical nursing practice. It may assist nursing managers in identifying gaps in foot care competence across departments and inform targeted educational interventions. In addition, the instrument has potential applicability in hospitals, community settings, and long-term care facilities, providing quantitative evidence to support geriatric foot health management and care process optimization. Integration with digital platforms may further enhance efficiency and scalability.

## 4. Limitations

This study has three limitations. First, participants were recruited from two tertiary hospitals in Guizhou and Chongqing using convenience sampling, and test–retest reliability was assessed in a relatively small subsample of 20 participants. These factors may limit the generalizability of the findings and the robustness of the stability estimates. Second, the questionnaire was evaluated using cross-sectional data; therefore, its responsiveness to changes following educational or practice-based interventions could not be assessed. Third, principal component analysis with varimax rotation was used during exploratory factor analysis, and orthotists were not included in the expert consultation process. Although these methodological choices were considered appropriate in the context of this study, future research could incorporate alternative factor extraction methods and broader multidisciplinary expertise to further strengthen the questionnaire development process.

Future studies should validate the questionnaire in multicenter samples involving more diverse nursing populations and healthcare settings. Larger samples are also needed to further evaluate test–retest reliability and confirm the stability of the instrument. Longitudinal studies are warranted to examine the responsiveness of the questionnaire over time and its usefulness in evaluating geriatric foot care education and training initiatives.

## Supporting information

S1 File Geriatric foot care knowledge–attitude–practice questionnaire. (DOCX)
