# Supplementary material for: Development and psychometric validation of a knowledge–attitude–practice questionnaire on geriatric foot care among clinical nurses
Source: PLoS One. 2026 Aug 17;21(8):e0356470. doi: 10.1371/journal.pone.0356470 (PMC13480607; doi:10.1371/journal.pone.0356470)
Supplement: Supplementary file 1 [file pone.0356470.s001.docx]

**Knowledge–Attitude–Practice Questionnaire on Geriatric Foot Care for Clinical Nurses**

（一）Knowledge of Geriatric Foot Care Among Clinical Nurses

Instructions for Completing the Questionnaire:

This section is designed to assess clinical nurses’ knowledge of geriatric foot care. Each item is followed by three response options: “Yes,” “No,” and “I don’t know.” Please select the option that best reflects your actual level of knowledge regarding foot care.

| **Geriatric Foot Care Knowledge Items** | YES | NO | I don’t know |
| --- | --- | --- | --- |
| K1 Foot health assessment is required for older adults during hospitalization |  |  |  |
| K2 Corns and calluses are caused by prolonged abnormal pressure or friction |  |  |  |
| K3 Dry foot skin requires daily use of moisturizers, including between the toes |  |  |  |
| K4 Toenails should preferably be trimmed before washing or when the feet are dry |  |  |  |
| K5 The correct method of toenail trimming is to cut off the corners of the nail |  |  |  |
| K6 Cutting toenails too short may lead to ingrown toenails |  |  |  |
| K7 Fungal infections of the nail or skin should be treated promptly to prevent ulcers and secondary infections |  |  |  |
| K8 Flatfoot should be managed with appropriately sized shoes that provide arch support and heel stability |  |  |  |
| K9 Collapse of any of the three foot arches may result in foot problems |  |  |  |
| K10 The feet of patients with diabetes should be assessed daily |  |  |  |
| K11 Routine foot soaking is an appropriate skin care practice for patients with diabetes |  |  |  |
| K12 For patients with rheumatoid arthritis, footwear should provide adequate toe box height and width |  |  |  |
| K13 Reduced skin temperature in one foot may indicate vascular obstruction |  |  |  |
| K14 Older adults are advised to wear soft-soled shoes to prevent falls |  |  |  |
| K15 Feet tend to become wider and longer with age, making regular shoe size assessment important |  |  |  |
| K16 Adequate toe space requires the shoe interior to be 1–1.5 cm longer than the longest toe |  |  |  |
| K17 Shoes with shallow toe boxes can prevent toe deformities |  |  |  |
| K18 Five-toe socks can reduce interdigital maceration and prevent tinea pedis |  |  |  |
| K19 Corns and calluses are not influenced by the type of socks worn |  |  |  |

1. Attitudes Toward Geriatric Foot Care Among Clinical Nurses

Instructions for Completing the Questionnaire:

This section is designed to assess clinical nurses’ attitudes toward geriatric foot care. Each statement is followed by five response options: “Strongly disagree” “Disagree” “Neutral” “Agree” and “Strongly agree” Please select the option that best reflects your personal feelings.

| Geriatric Foot Care Attitude Items | Strongly disagree | Disagree | Neutral | Agree | Strongly agree |
| --- | --- | --- | --- | --- | --- |
| A1 I believe that nurses play an important role in geriatric foot health management |  |  |  |  |  |
| A2 I believe that the foot care needs of older adults are currently unmet |  |  |  |  |  |
| A3 I believe that foot pain and infections have the greatest impact on older adults |  |  |  |  |  |
| A4 I believe that older adults with chronic diseases (e.g., diabetes) are priority populations for foot care |  |  |  |  |  |
| A5 I believe that shoe size assessment and footwear selection are essential for geriatric foot care |  |  |  |  |  |
| A6 I believe that geriatric foot health management requires multidisciplinary collaboration |  |  |  |  |  |
| A7 I believe that providing foot care health education to older adults is necessary |  |  |  |  |  |
| A8 I believe that foot care can promote foot health in older adults |  |  |  |  |  |
| A9 I believe that disease characteristics and clinical symptoms must be considered when providing foot care |  |  |  |  |  |

1. Practices of Geriatric Foot Care Among Clinical Nurses

Instructions for Completing the Questionnaire:

This section is designed to assess clinical nurses’ practices related to geriatric foot care in their daily work. Each statement is followed by five response options. Please select the option that best reflects your actual practice:

Never (performed 0 times per month)

Occasionally (performed 1–3 times per month)

Sometimes (performed 4–6 times per month)

Often (performed 7–10 times per month)

Always (performed more than 10 times per month)

| Geriatric Foot Care Practice Items | Never | Occasionally | Sometimes | Often | Always | |
| --- | --- | --- | --- | --- | --- | --- |
| P1 I assess the foot skin of older adults, including between toes and heels |  |  |  |  |  | |
| P2 I guide or assist older adults in maintaining foot hygiene, including drying between toes after washing |  |  |  |  |  | |
| P3 I guide or assist older adults in applying moisturizer after foot washing to prevent dryness |  |  |  |  |  | |
| P4 I guide or assist older adults in managing corns or calluses appropriately |  |  |  |  |  |  |
| P5 I assess the toenail condition of older adults |  |  |  |  |  |  |
| P6 I guide or assist older adults in trimming toenails correctly (straight cut, level with the toe, smoothing edges with a file) |  |  |  |  |  | |
| P7 When mild ingrown toenails occur, I know how to place cotton under the nail edge to relieve discomfort |  |  |  |  |  | |
| P8 When older adults have fungal nail infections, I instruct them to use antifungal medication as prescribed |  |  |  |  |  | |
| P9 When examining feet, I assess pedal pulses, skin temperature, and sensation |  |  |  |  |  | |
| P10 I instruct older adults with conditions such as diabetes to wash their feet properly and follow precautions |  |  |  |  |  | |
| P11 I guide older adults to perform foot self-examinations |  |  |  |  |  | |
| P12 When serious foot problems are identified, I seek multidisciplinary collaboration |  |  |  |  |  | |
| P13 I recommend that older adults with diabetes or osteoarthritis attend podiatry clinics for foot health assessment |  |  |  |  |  | |
| P14 I guide older adults in selecting appropriate footwear and socks, including material, width, depth, and heel height |  |  |  |  |  | |
| P15 I guide and assist older adults in using foot orthoses or supportive footwear when necessary |  |  |  |  |  | |
| P16 I advise older adults not to wear socks with excessive seams or holes |  |  |  |  |  | |
| P17 I encourage older adults to perform foot function exercises |  |  |  |  |  | |
| P18 I encourage older adults to stand after prolonged sitting (≥1 hour) |  |  |  |  |  | |
| P19 I encourage older adults, especially those with diabetes, to stop smoking |  |  |  |  |  | |
| P20 I regularly provide foot care health education to older adults or their caregivers |  |  |  |  |  | |
